# A systematic review and meta-analysis of the executive function-health behaviour relationship

**DOI:** 10.1080/21642850.2019.1637740

**Published:** 2019-07-09

**Authors:** Kara Gray-Burrows, Natalie Taylor, Daryl O’Connor, Ed Sutherland, Gijsbert Stoet, Mark Conner

**Affiliations:** a School of Dentistry, University of Leeds, Leeds, UK; b Cancer Council NSW, Sydney, Australia; c School of Psychology, University of Leeds, Leeds, UK; d Department of Psychology, University of Essex, Colchester, UK

**Keywords:** Executive function, health-protective behaviour, health-damaging behaviour, moderators, meta-analysis

## Abstract

**Objective:**

This study provides the first comprehensive meta-analysis of the relationship between executive function (EF) and performance of health behaviours in healthy populations.

**Method:**

Electronic databases (MEDLINE, Embase, PsycINFO, Web of Science) were searched, and forward and backward citation tracking was undertaken to identify articles investigating the relationship between EF and health behaviour. Studies were eligible if they examined the direct correlational relationship between EF and health behaviour in healthy populations, were available in English and published in peer-reviewed journals in any year.

**Results:**

Sixty-one articles covering 65 tests were included in a random effects meta-analysis. Several moderators were assessed, including: the type, and addictiveness of the health behaviour; the type of EF measure; study design, and sample characteristics. Overall EF had a significant, but small correlation with health behaviour; EF was significantly positively associated with health-protective behaviours and significantly negatively associated with health-damaging behaviours. There was considerable heterogeneity in the observed effect sizes, but this was not explained by the examined moderators.

**Conclusions:**

Although the meta-analysis indicates a significant effect for EF on health behaviour, effect size is small. Due to the complex nature of EF, more research is required to further elucidate the relationship between EF and health behaviour in its entire conceptualization.

## Introduction

The link between health behaviours and morbidity and mortality has long been of interest (e.g. Alameda County study; Belloc & Breslow, 1972). Subsequently, considerable research effort has explored the determinants of such health behaviours with a focus on behaviour-specific health cognitions (e.g. intentions, self-efficacy; Conner & Norman, 2005) and individual personality traits (e.g. conscientiousness; see Bogg & Roberts, 2004). Recently researchers have tested executive function (EF) as a further construct to predict variability in health behaviours.

EF is an umbrella term for the higher order ‘top-down’ cognitive processes that allow the co-ordination of thought and action, which have been measured using behavioural and neurophysiological methods in both humans and animals (Stoet & Snyder, 2009). Although the biological underpinnings of EF are complex, at a basic neuroanatomical level, EF is linked to the prefrontal cortex and anterior cingulate cortex (van Veen & Carter, 2006). EF is multifaceted in nature and includes an array of functions relevant to the execution of goal-directed behaviour. Funahashi (2001), for example, describes EF as the ‘coordinated operation of various processes to accomplish a particular goal in a flexible manner’ (p. 147). The four key domains of EF are *inhibition* (exerting deliberate control over pre-potent responses), *shifting* (cognitive flexibility in switching between tasks/mental sets), *updating* (monitoring and updating working memory) and *planning* (Miyake & Friedman, 2012; Miyake, Friedman, Emerson, Witzki, & Howerter, 2000). Proficiency in each of these different domains has been suggested to be an important determinant of the performance of health behaviour (Dohle, Diel, & Hofmann, 2018). For example, the ability to inhibit undesirable responses might help individuals avoid performing health-damaging behaviours, such as smoking. Similarly, the EF function ‘planning ahead’ might be conducive to successful engagement with health-protective behaviours, which require actions to maintain health, such as healthy eating and physical activity. Individuals high in EF are assumed to be generally more likely to successfully initiate behaviour change and maintain that change in pursuit of their goals (Allan, 2008; Allan, Johnston, & Campbell, 2011; Brega, Grigsby, Kooken, Hamman, & Baxter, 2008; Hall, Dubin, Crossley, Holmqvist, & D'Arcy, 2009; Wong & Mullan, 2009).

Early research on the relationship between EF and health behaviours focused on vulnerable populations (e.g. the elderly; Brega et al., 2008). A more recent focus has been on these relationships in healthy populations (Allan, Sniehotta, & Johnston, 2013; Allom, Mullan, & Sebastian, 2013; Hall, Fong, & Epp, 2014; Pieters, Burk, Van der Vorst, Engels, & Wiers, 2014; Salemink & Wiers, 2014; Sharbanee, Stritzke, Wiers, & MacLeod, 2013). Despite this growing body of literature, to date, there are no meta-analytic reviews of the relationship between EF and health behaviour in healthy populations, particularly on those free from mental health problems or brain injury. de Ridder, Lensvelt-Mulders, Finkenauer, Stok, and Baumeister (2012) undertook a meta-analysis exploring the relationship between self-control and behaviour, including eating and addictive behaviours, but this only focused on the influence of one component of an EF-related construct to a small number of health behaviours. Both EF and health behaviour are complex, thus bringing clarity to where the relationships lie between the two and the nature of these relationships, particularly regarding what specific components of EF are beneficial, or indeed, detrimental to specific health behaviours is of vital importance to future individual-level behaviour change interventions or public health campaigns. Assessing this first within healthy populations will allow us to understand and use this information universally, which can then be hopefully used as a baseline, and tailored to further explore the nuances of clinical populations.

In examining the impact of EF on health behaviour we explored a number of moderators linked to the behaviour, EF measure, study design and sample. An important initial distinction that needs to be made is regarding the difference between health-protective and health-damaging behaviours. Health-protective behaviours are health-enhancing behaviours, which individuals are encouraged to perform to protect their health; whereas health-damaging behaviours are health-harming behaviours, which individuals are encouraged to perform less to reduce any detrimental impact on their subsequent health. Recent research has emphasized the importance of different predictors for the two types of health behaviour (e.g. Conner, McEachan, Lawton, & Gardner, 2017). As noted above EF is assumed to be positively related to protecting the self, i.e. increasing the performance of health-protection behaviours, but the decreasing performance of health-damaging behaviours. Our analyses both examined the extent to which EF was associated with health behaviour in the predicted direction and also whether the effects were different in across health-protective and health-damaging behaviours.

In relation to characteristics of the behaviour, we examined the impacts of EF for various different health-protective (e.g. physical activity) and health-damaging (e.g. alcohol use) behaviours. We also examined the moderating effects of the addictiveness of behaviours, and whether the behaviour measures were objective or self-report. The power of EF to predict addictive (e.g. alcohol, smoking, drug use) health-damaging behaviours may be attenuated compared to non-addictive (e.g. snack consumption) health-damaging behaviours. In relation to the measurement of health behaviours, objective measures are generally to be preferred, but are less well predicted than self-report measures (e.g. Armitage & Conner, 2001). Nevertheless, assessing both is vital to truly gauge the validity of the EF-behaviour relationship.

In relation to types of EF measures, we examined the moderating effects of the specific type of EF measure used, and whether the EF measures were objective or self-reported. A variety of EF measures have been employed in this domain including measures tapping cognitive flexibility, planning, response inhibition, working memory and measures that tap combinations of these measures. In line with Miyake et al. (Miyake et al., 2000; Miyake & Friedman, 2012) *Shifting, Inhibiting and Updating* domain structure of EF; tasks of cognitive flexibility reflect the *shifting* domain and include tasks that require an individual to switch between mental sets (e.g. the Trail-Making task), whereas response inhibition reflects the domain of *inhibition* and thus includes any task (e.g. the Stop signal task) where an individual must actively inhibit a pre-potent response. Working memory reflects the *updating* domain and includes tasks such as the Operation Span task where information must be stored and actively worked on. A recent addition to structure of EF is planning, which reflects any task where an individual must pre-plan a strategy to successfully complete the task (e.g. Tower of London; Shallice, 1982). Response inhibition measures are assumed to be particularly important in reducing health risk behaviours such as snacking (Allan et al., 2011; Hall, 2012) and alcohol consumption (Christiansen, Cole, Goudie, & Field, 2012; Colder & O'Connor, 2002). While planning (Allan et al., 2013; Hall et al., 2008) and working memory (Hofmann, Gschwendner, Friese, Wiers, & Schmitt, 2008; Houben, Wiers, & Jansen, 2011; Romer et al., 2011) measures have been explored as predictors of both health-protective and health risk behaviours. We examined whether there was evidence that response inhibition, cognitive flexibility, working memory, planning or combination measures of EF were more predictive of health behaviours. The effect of using objective versus self-reported EF measures was also assessed. Traditionally, objective measures of EF have been mainly utilized, however, self-report measures, such as the Dysexecutive questionnaire have recently been used in this type of research and potentially offer a more ecologically valid measure. On the other hand, objective measures may be less susceptible to bias and lack of awareness effects.

In relation to aspects of the methodology we examined whether cross-sectional or longitudinal study designs were used, sample characteristics, and the risk of bias. Cross-sectional designs, where all variables are measured in the same session, are more open to consistency biases (Armitage & Conner, 2001) and may overestimate effects of EF on health behaviours compared to longitudinal designs (EF measured before health behaviour). For sample characteristics, we examined the effects of sample age and whether the sample was from the clinical or general population. EF has a developmental trajectory that means EF develops over time and does not reach full functionality until an individual’s early twenties (Eshel, Nelson, Blair, Pine, & Ernst, 2007; Lyon & Krasnegor, 1996; Romine & Reynolds, 2005) with evidence of decline in later years (Robbins et al., 1998). Hence, we anticipated that the effects of EF might be weaker in children/adolescent groups compared to adult groups (those likely to be experiencing a decline in EF were not examined here; i.e. all studies with adults reported no cognitive declines in their sample). Given the review focuses on non-cognitively impaired groups we did not expect strong differences between clinical and non-clinical samples.

In summary, the present manuscript provides the first meta-analysis to distinctly assess several over-arching components of EF (i.e. inhibition, updating, shifting, planning) and several health behaviours, including both health-protective and health-damaging behaviours, in healthy populations. The current research is underpinned by the PRISMA checklist (See Supplementary materials), and fills a significant gap in the literature. Moreover, we examine not only the overall effect size for the relationship between EF and health behaviour, but also the effects of various potential moderators.

## Method

### Search and inclusion/exclusion criteria

**Figure 1.**
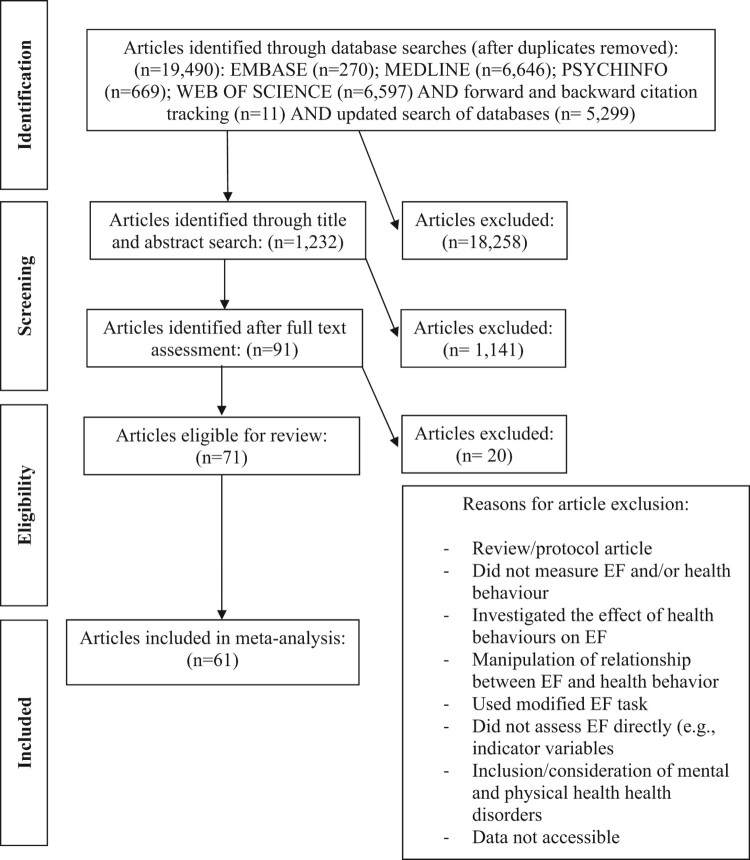
Meta-analysis search strategy and screening process.

A range of search strategies were employed to obtain relevant studies. First, four electronic databases (Web of Science, PsycINFO, MEDLINE and Embase) were searched between October and November 2014 and updated in March 2018 to identify any new papers that had been published during the time that had elapsed since the first search, for peer-reviewed journal articles available in English with human participants published in any year (See Supplementary Material 4 for an example of the search strategy). Search terms were kept as broad as possible to ensure all relevant articles were identified; however, additional specific health behaviour terms were included upon the recommendation of experts in the field and based upon the findings of an initial scoping review. Second, forward and backward citation tracking was undertaken on all articles included within the review to identify any additional relevant studies (see Figure 1). Studies were excluded if: (i) they did not measure EF or used a modified version of an EF measure (i.e. where it was modified to include the health behaviour in question, for example, food-specific or alcohol-specific, as the current review assessed the relationship between general EF function and health behaviours); (ii) they did not measure health behaviour (i.e. any behaviour that has a short and/or long term impact on health, which are, in part, within the individuals control; and for the purpose of this review, measured directly); (iii) the study attempted to manipulate the relationship between EF and health behaviour, as the present review aimed to examine the simple direct relationship between EF and health behaviour, as understanding this will allow enhanced tailoring of subsequent experimental studies; (iv) participants had incurred head trauma or had mental health problems, including depression, anxiety and conduct disorders (see Suchy, 2009 for evidence of the impact on EF); (v) participants suffered from any physical health condition, with the only exception being if the study was related to medication adherence. Studies were included if they used any measure of EF plus health behaviour and reported the relationship between the two plus the sample size. Where correlations between EF and health behaviour were not reported authors were contacted requesting this data.

### Coding

Effect sizes were coded as positive if higher levels of EF were associated with greater performance of the health behaviour and negative if higher levels of EF were associated with the decreased performance of the health behaviour. However, to obtain an overall effect size across all health behaviours we reversed this coding for health-damaging behaviours. Such that the coding indicated whether the effects were in the predicted direction (i.e. a positive relationship between EF and health-protective behaviours and a negative relationship between EF and health-damaging behaviours). Where appropriate, effects were meta-analyzed within a study (e.g. where effects were reported for several health behaviours within a single study, effects were averaged across behaviours) and the appropriate effect sizes for the study were used in subsequent analyses. For the moderator analyses, studies were coded by health behaviour, EF measure, addictiveness, study design, and sample. In order to maximize the number of analyses available for the analyses without biasing the findings, we used all the relevant effect sizes reported within a study. For example, where a study reported effects separately for different levels of a moderator (e.g. for snacking consumption and alcohol use) these different values were each used in the analyses, but the sample size was reduced appropriately (Borenstein, Hedges, Higgins, & Rothstein, 2005).

### Behaviour moderators

Initially, each study was coded as examining a health-protective or health-damaging behaviour. Studies that examined both health-protective and health-damaging behaviours without providing separate effect sizes for each were excluded from this analysis.

In relation to *behaviour type*, studies were coded into fruit and vegetable consumption, medication adherence, physical activity, sleep, sun protection, alcohol use, drug use, smoking or snack consumption. All studies testing effects across multiple health behaviours reported the separate effects.

In relation to *addictiveness of behaviours*, we coded protection behaviours into addictive (i.e. alcohol, smoking, drugs) or non-addictive (i.e. snack consumption) behaviours.

Method of behavioural measurement was coded into objective and self-report. Objective measures of health behaviour included actual consumption, medication adherence as measured by medication event monitoring systems (MEMS) and physical activity as measured by number of steps or exercise class attendance. On the other hand, self-report measures included the Time Line Follow Back questionnaire to assess alcohol consumption (e.g. Henges & Marczinski, 2012).

### EF moderators

EF measure type was coded into response inhibition (e.g. Stroop task), cognitive flexibility (e.g. Wisconsin Card Sorting Task (WCST)), working memory (e.g. computation span task), planning (e.g. Tower of Hanoi), or combined measures (e.g. DEX) based on the nature of the tasks used within each study using the domains outlined by (Miyake et al., 2000; Miyake & Friedman, 2012). Other measures of EF, particularly self-report measures, may combine a number of these elements; for example, the DEX includes questions pertaining to response inhibition and planning, thus as these separate EF functions cannot be accurately teased apart such measures were categorized as combined measures. Studies testing effects across multiple EF measures that did not report separate effects were classified as ‘combined’. EF measures were also coded into objective (e.g. Go/No-go task, Stop-signal task, operation span task, Tower of London/Hanoi, Stroop task, Trail-making task, Verbal fluency, WCST, task switching) and self-reported (e.g. DEX, Inhibitory Control Scale).

### Study design moderators

*Study design* was coded into cross-sectional (all measures taken at the same time point); or longitudinal (health behaviour measured at a separate and later time point to EF measures).

### Sample characteristics

*Sample age* was coded into children/adolescents or student/adults. Studies with samples that fell into both categories (i.e. comprised of both children and adults) that did not report separate effects were excluded from the analysis. *Sample type* was coded into non-clinical (healthy populations with no diagnosed physical or mental illness) or clinical (entire sample comprised of individuals diagnosed with a physical illness/disorder). Again, studies with samples that fell into both categories (i.e. comprised of both healthy population and clinical groups) that did not report separate effects were excluded from the analysis.

### Quality

*Risk of bias* was coded into low or no risk (deemed as low risk for all assessed criteria), or potential risk (deemed as having a potential risk of bias for one or more of the assessed criteria) using a modified version of a quality assessment tool (based on the Higgins, Altman, & Sterne, 2011 tool used by Ntoumanis, Ng, Barkoukis, & Backhouse, 2014, see Supplementary Material 2). Coding was conducted by three individuals trained to PhD level. Percentage agreement was 72% and disagreements were resolved through discussion. However, only one study was deemed as no or low risk for all assessed criteria (Ickmans et al., 2013) precluding further analysis.

### Data synthesis

Each correlation coefficient (including where studies provided multiple correlations between EF measures and health behaviours) was rounded to two decimal places. Random effects meta-analysis was then conducted using the Comprehensive Meta-analysis programme (Borenstein et al., 2005) with effect size estimates weighted by sample size. Number of studies (*k*), total sample size (*n*), mean effect sizes (*r_+_*) and 95% confidence intervals (*95%CI*) were computed along with heterogeneity estimates (*Q* statistic; *I^2^* value). Significant *Q* values were indicative of significant heterogeneity. The *I^2^* statistic was used to quantify the degree of heterogeneity (*I^2^* values of 25%, 50% and 75% indicate low, moderate and high levels of heterogeneity, respectively; Higgins, Thompson, Deeks, & Altman, 2003). In addition, Egger, Davey Smith, Schneider, and Minder (1997) regression test and the Duval and Tweedie (2000) trim and fill procedure were used to identify potential publication bias. Moderator analyses were undertaken using random effects subgroup analyses. Variance between studies was expected to be consistent across subgroups, and thus the heterogeneity variance within each subgroup (τ^2^) was estimated by a single value collapsed across subgroups (Borenstein et al., 2005). Statistical significance of each moderator was assessed using *Q*-tests analogous to analysis of variance, such that a significant between-groups *Q* indicates that the effect size differs significantly as a function of the moderator.

Analyses examined effects across health-protective and health-damaging behaviours separately and moderators of these effects. We also examined effects across all behaviours (after reversing the effects for studies classified as examining health-damaging behaviours).

## Results

### Study characteristics

Sixty-one articles (containing *k* = 65 independent analyses, *N* = 14,496) were included in the meta-analysis (Supplementary Table 1 and Supplementary material 3 provide a full list of included studies and study details). Sample sizes ranged from 27 to 2,230 participants. Most studies included both males and females, although six only included females (Giancola & Mezzich, 2003; Hofmann et al., 2008; Hofmann, Friese, & Roefs, 2009; Ickmans et al., 2013; Patrick, Blair, & Maggs, 2008; Stilley, Bender, Dunbar-Jacob, Sereika, & Ryan, 2010) and one included only males (Friese, Bargas-Avila, Hofmann, & Wiers, 2010). More studies examined health-damaging compared to health-protective behaviours (Table 1). Alcohol consumption was the most frequently examined behaviour (*k* = 23). There were more addictive (*k* = 29) compared to non-addictive (*k* = 7) health-damaging behaviours and considerably more studies utilizing self-reported (*k* = 44) compared to objectively (*k* = 8) measured health behaviours. Response inhibition measures were the most commonly used EF measure (*k* = 31). Most studies used objective EF measures; for instance, all but one of the studies examining health-protective behaviours used such measures. Longitudinal designs were more commonly used in studies of health-protective behaviours (*k* = 14), while cross-sectional designs were more commonly used in studies of health-damaging behaviours (*k* = 23). There were also more studies on student/adults compared to children/adolescents (for instance, all of the studies examining health-protective behaviours were with student/adult samples; see Table 1).

**Table 1.** Number of studies, sample sizes, effect size plus 95% CI and heterogeneity effects (Q and I^2^) for EF in health-protective and health-damaging behaviours.

	Health-protective behaviours						Health-damaging behaviours					
	k	N	r+	95%CI	Q	I^2^	k	N	r+	95%CI	Q	I^2^
Overall effect	21	9305	.090*	.031, .148	75.7	73.6	50	12753	−.044*	−.077, −.011	115.6	57.6
*Health Behaviour Moderators*												
*Behaviour type*												
Fruit/veg consumption	6	3783	.062	−.064, .187	7.2	30.5						
Medication adherence	3	479	.176	−.017, .356	10.0	80.1						
Physical activity	6	2708	.085	−.051, .218	8.2	33.4						
Sleep	4	1492	.110	−.040, .256	43.7	93.1						
Sun protection	2	387	−.140	−.339, .071	2.6	61.0						
Alcohol							33	5854	−.025	−.068, .018	67.2	52.3
Drugs							5	589	−.086	−.205, .036	7.9	49.6
Smoking							11	4740	−.042	−.108, .024	26.5	62.3
Snack consumption							10	1047	−.135*	−.214, −.053	17.2	47.7
Substance abuse							2	523	−.110	−.250, .035	4.2	76.3
Addictive behaviours							40	6283	−.044*	−.081, −.007	107.2	63.6
Non−addictive behaviours							10	678	−.125*	−.207, −.042	18.4	51.0
Objective behaviour measure	6	2014	.049	−.067, .164	10.9	54.1	3	297	−.155*	−.301, −.003	4.8	58.0
Self−report behaviour measure	16	4707	.049	−.017, .114	68.1	78.0	47	12106	−.047*	−.082, −.011	127.2	63.8
*EF Moderators*												
*EF measure type*												
Cognitive flexibility	7	1271	.074	−.048, .193	18.9	68.3	5	1724	−.030	−.131, .071	3.3	0.0
Response inhibition	14	2909	.098*	.014, .180	49.2	73.6	33	4601	−.025	−.068, .018	64.1	50.0
Planning	7	607	−.040	−.162, .084	1.6	0.0	7	483	−.022	−.133, .089	6.3	5.1
Combined measure	5	947	.127	−.010, .259	11.3	64.6	6	1379	−.149*	−.233, −.063	21.0	76.1
Working memory	4	997	.126	−.042, .287	1.9	0.0	20	4218	−.028	−.079, .022	23.2	18.3
Objective EF measure							48	11921	−.045	−.081, −.009	124.7	62.3
Self−reported EF measure							4	456	.097	−.243, .053	11.1	72.9
*Study design*												
Cross-sectional design	2	605	.041	−.138, .217	0.1	0.0	28	3644	−.067*	−.116, −.018	84.5	68.1
Longitudinal design	19	6119	.061	−.003, .125	80.4	77.6	22	8759	−.049*	−.097, .000	42.8	51.0
*Sample Moderators*												
Child/adolescent							15	8390	−.059*	−.109, −.008	36.0	61.2
Student/adult							34	3820	−.043*	−.086, .001	74.1	55.4
Non-clinical sample	16	5769	.052	−.018, .122	71.6	79.0						
Clinical sample	3	479	.118	−.061, .289	8.3	75.8						

*p < .05.

### Overall effects

There was a significant, but small (Cohen, 1992) positive effect between EF and health-protective behaviours (*r*_+_ = .090, 95%CI = .031 to .148, *p =* .003, *k *= 21, *N* = 9,305) and a significant, but small negative effect between EF and health-damaging behaviours (*r*_+_ = −.044, 95%CI = −.077 to −.011, *p *= .009, *k *= 50, *N* = 12,753) (Table 1). The effect sizes for health-protective and health-damaging behaviours were significantly different from one another, *Q*(1) = 15.04, *p* < .0001. This justified subsequent analyses focusing on health-protective and health-damaging behaviours separately. Nevertheless we also confirmed that, after reversing the coding for health-damaging behaviour studies, there was a small significant relationship between EF and health behaviour overall (*r*_+_ = .044, 95%CI = .000 to .088, *p *= .050, *k *= 65, *N *= 14,496), although there was considerable heterogeneity across studies (*Q*(64) = 387.9, *p* < .0001; *I*^2^ = 83.5%). This indicates that EF was associated with health behaviour in the predicted direction (i.e. increased the performance of health-protective behaviours, but decreased the performance of health-damaging behaviours).

There was also considerable heterogeneity in the overall effects for both health-protective (*Q*(20) = 75.7, *p* < .0001; *I*^2^ = 73.6%) and health-damaging (*Q*(49) = 115.6, *p* < .0001; *I*^2^ = 57.6%) behaviours suggesting the value of testing for the effects of potential moderators within each behaviour type. Egger’s regression test did not reveal significant asymmetry for health-protective (*p* = .07, two-tailed) or health-damaging (*p* = .45, two-tailed) behaviours. The funnel plots did not support strong evidence of bias for either health-protective or health-damaging behaviours (Sterne et al., 2011; see Supplementary Materials for plots). However, the Trim and Fill method (Duval & Tweedie, 2000) suggested trimming 8 studies from the left of the mean for health-protective behaviours and 6 studies from the left of the mean for health-damaging behaviours (0 studies were trimmed from the right of the mean for health-protective or health-damaging behaviours). Trimming reduced the effect size for health-protective behaviours (*r*_+_ = .009, 95%CI = −.058 to .075), but did not substantively change the effect size for health-damaging behaviours (*r*_+_ = −.062, 95%CI = −.095 to −.029). Together these findings suggest the influence of publication bias in the meta-analysis can be designated as modest rather than severe, but had more influence on the results for health-protective compared to health-damaging behaviours.

#### Health-protective behaviour moderators

Within the health-protective behaviours, examination of potential moderators indicated that there were no significant differences based on health behaviour type, *Q*(4) = 5.32, *p* = .26 (Table 1). Finally, there were no significant differences between studies using objective and self-report measures of health behaviour, *Q*(1) = 0.00, *p* = 1.00, with effects non-significant for both types of measure.

In relation to EF measure moderators, there were no significant differences attributable to EF measure type, *Q*(4) = 4.50, *p* = .34, although effects were significant for studies using cognitive flexibility and response inhibition measures, but not planning, working memory measures, or combined measures (Table 1). In relation to study design, there were no significant differences between cross-sectional and longitudinal studies, *Q*(1) = 0.04, *p* = 1.00, with effects non-significant for both types of design. Finally, in relation to sample moderators, there were no significant differences between studies using non-clinical and clinical samples, *Q*(1) = 0.45, *p* = 0.50, with effects non-significant for both types of sample.

#### Health-damaging behaviour moderators

Within the health-damaging behaviours, examination of potential moderators indicated that there were no significant differences based on health behaviour type, *Q*(4) = 6.47, *p* = .26 (Table 1). There were also no effects associated with addictive versus non-addictive behaviours, *Q*(1) = 3.07, *p* = .08, although EF was significantly negatively associated with both addictive and non-addictive behaviours. Finally, in relation to health behaviour measure moderators, there were no significant differences between studies using objective and self-report measures of health behaviour, *Q*(1) = 1.87, *p* = 0.17, with effects significant and negative for both types of measure.

In relation to EF measure moderators, there were no significant differences attributable to EF measure type, *Q*(4) = 7.00, *p* = .14, although effects were significant for studies using combined measures, but not cognitive flexibility, response inhibition, planning, or working memory measures. There were also no significant differences between objective versus self-report measures of EF, *Q*(1) = 0.45, *p* = .50, although effects were significant for studies using objective measures, but not for studies using self-report measures (Table 1).

In relation to study design, there were no significant differences between cross-sectional and longitudinal studies, *Q*(1) = 0.26, *p* = 0.61, with effects significant for both types of design. Finally, in relation to sample moderators, there were no significant differences between studies using child/adolescent versus student/adult samples, *Q*(1) = 0.23, *p* = 0.63, with effects significant for the former, but not the latter. There were also no significant differences between studies using non-clinical and clinical samples, *Q*(1) = 0.00, *p* = 0.98, with effects significant for the former, but not the latter.

## Discussion

This is the first meta-analytic review to summarize the published research examining the correlation between EF and health behaviours in healthy populations (ones free from mental health problems/brain injury and/or physical health conditions except for medication adherence studies). A total of 61 studies reporting 65 tests of this relationship were included in the meta-analysis. Overall, the results indicated that EF had a statistically significant, but small sized, effect on health behaviour in the predicted direction, i.e. better EF was associated with greater engagement in health-protective behaviours and less engagement in health-damaging behaviours.

Subsequent analyses revealed that, as expected there were significant, but small positive effects of EF on health-protective behaviours and significant, but small negative effects of EF on health-damaging behaviours. Egger’s regressions and trim and fill analyses and funnel plots suggested that neither of these effects were likely to be strongly biased by unpublished studies, although trimming studies from the analyses of health-protective behaviours reduced the effect size to non-significance.

Although the meta-analysis indicated considerable heterogeneity in the findings there were no significant moderation effects. In relation to both health-protective and health-damaging behaviours, no significant moderation effects emerged in relation to the type of health behaviour, types of EF measures used, aspects of the study design or sample employed. We conclude that, based on the existing evidence, the relationship between EF and health-protective and health-damaging behaviours is invariant across these moderators. However, some caution should be exercised in this conclusion given the small number of studies in some categories.

Despite not finding evidence of significant moderating effects for type of EF measure it is noteworthy that among health-protective behaviours, only studies using response inhibition measures of EF showed significant positive effects on health behaviour. In contrast, among health-damaging behaviours, only studies using a combined measure of EF showed significant negative effects on health behaviour. Future research might usefully explore whether such effects are replicated in prospective studies. This is important given that a priori we might have expected planning aspects of EF to be particularly associated with greater performance of health-protective behaviours and response inhibition to be particularly associated with reduced performance of health-damaging behaviours.

Furthermore, it must be acknowledged that the conceptualisation of EF is extremely complex. This review aimed to explore EF at its most general level in line with most of the studies included within the current review, though there are notable exceptions (e.g. Allan et al., 2011). For instance, performance on EF measures is heavily influenced by momentary influences, such as tiredness, caffeine, emotional state, and motivation. Therefore, how EF influences behaviour depends on the context, such that a lack of controlled processing could leave people more reactive to the environment and habit, which could lead people to act in ways that are more or less harmful to the self. Indeed, it is entirely plausible that in some instances even high-level EF could be detrimental to health behaviour (Allan & Allan, 2013; Hofmann, Schmeichel, & Baddeley, 2012; Patrick et al., 2008). In addition, it is important to remember that the relationship between EF and health behaviour is not unidirectional, but bidirectional, with changes in one having an impact on the other (Allan, McMinn, & Daly, 2016). Moreover, research suggests that self-report and objective measures of self-control do not measure the same aspects, but instead reflect differences in trait and specific self-control processes (Allom, Panetta, Mullan, & Hagger, 2016)

### Limitations

The main limitation of the current meta-analysis is the lack of studies in a number of areas that either precluded an examination of moderating variables or limited the power of such analyses. In relation to the former it is notable that no studies have examined the EF-health-protective behaviour relationship in children/adolescents. A related weakness is that the lack of studies precluded further examination of moderators hierarchically beyond examining the effects in health-protective and health-damaging behaviours. For example, too few studies precluded an examination of differences by sample within different designs. A third limitation is the modest power of the current moderators to explain the heterogeneity in effect sizes. A final limitation was the fact that the study was not pre-registered.

### Conclusions

The present meta-analysis indicated that EF has a statistically significant, but small sized overall effect on health behaviour (increasing performance of health-protective behaviours and decreasing performance of health-damaging behaviours). This overall effect size is heterogeneous and only partially explained by the moderators examined here in relation to characteristics of the behaviour, the measures, and the methodology. Further studies testing the relationship between EF and health behaviours are required. Such work can usefully inform the growing number of studies that manipulate EF variables as a means to produce health behaviour change at the level of the individual (Houben & Jansen, 2011; Houben, Havermans, Nederkoorn, & Jansen, 2012; Houben et al., 2011). Furthermore, by gaining greater understanding of the complex individual-level factors that influence health behaviour, we can subsequently explore how these interact with the social and physical environment, such that future health-promoting public health interventions and campaigns are tailored to take into account these potential barriers and enablers to health behaviour.

## Supplementary Material

Supplemental Material
