# Supplementary material for: A systematic review and meta-analysis of the executive function-health behaviour relationship
Source: Health Psychol Behav Med. 2019 Jul 9;7(1):253–68. doi: 10.1080/21642850.2019.1637740 (PMC8114370; doi:10.1080/21642850.2019.1637740)
Supplement: Supplemental Material [file RHPB_A_1637740_SM1348.docx]

On-line Supplementary Materials

Supplementary Figure 1. *Funnel plot of standard errors by Fisher’s Z effect size for health-protective behaviour studies*

Supplementary Figure 2. *Funnel plot standard errors by Fisher’s Z effect size for health-damaging behavior studies*

Supplementary Table 1: *Summary of studies included in review.*

|  | Sample and design | EF measures | Health behaviour/type of measure | Original effect sizes^[[1]](#footnote-1)^ | Risk of bias |
| --- | --- | --- | --- | --- | --- |
| Albertella et al. (2017) | Cross-sectional, n=66 undergraduate students, aged 18 – 31 (mean age 19.2) | Goal-directed selective attention (GDSA) task | Smoking, alcohol consumption, and drug use - Self-report | Smoking (number days/month):  r = .07  Alcohol consumption (number days/month)  r = -.08  Illicit drug use (lifetime use, total types):  r = -.09 | Potential |
| [Allan et al. (2013)](#_ENREF_4) | Study 1: Prospective, n=72 students; mean age 19.8 | ‘Zoo Map’ task | Snacking behaviour – Self-report | r =.19 | Potential |
| [Allan et al. (2011)](#_ENREF_3) | Study 1: Prospective; n=50 students (49 included in analysis); mean age 22  Study 2: Correlational; n=52 students; mean age 21 | Study 1: GNG, Tower Task, Verbal Fluency Task, Trail Making Task and the DEX  Study 2: GNG and the Stroop Task | Study 1: Fruit, vegetable and snack consumption – Self-report  Study 2: Snack consumption – Self-report | Study 1:  Fruit & vegetable consumption:  r = .30 (DEX)  r = .03 (GNG)  r = .14 (Tower task)  r = .20 (Verbal fluency)  r = .25 (Trail-Making)  Snack consumption  r = .36 (DEX)  r = -.34 (GNG)  r = .33 (Tower task)  r = .12 (Verbal fluency)  r = .23 (Trail-Making)  Study 2:  r = .30 (Stroop)  r = .18 (GNG) | Potential |

| [Allan et al. (2010)](#_ENREF_2) | Empirical; n=62 students intending to avoid calorific snacks, mean age 20.4 | Stroop Task, Tower Task and a Fluency Task | Chocolate consumption - Objective | r = .48 (Stroop)  r = .39 (Fluency)  r = .22 (Tower task) | Potential |
| --- | --- | --- | --- | --- | --- |
| [Allom et al. (2013)](#_ENREF_6) | Study 1: Prospective; n=218 students (209 included in analysis; ages 16-45 (mean 20.06)  Study 2: Prospective, n=227 students (178 included in analysis), ages 17-44 (mean 19.41) | TOL and WCST | Sun protection behaviours – Self-report | Study 1:  r = -.13 (TOL)  r = .01 (WCST)  r = .07 (IGT)  Study 2:  r = -.01 (TOL)  r = .43 (WCST)  r = -.01 (IGT) | Potential |
| Allom & Mullan (2014) | Prospective, n=115 (Note: 5 excluded from Stroop analysis) students; mean age 19.79 | Stroop interference task, Stop-signal task, n-back, operation span task | Saturated fat, and fruit and vegetable intake – Self-report | Saturated fat intake:  r = .30 (Stroop)  r = .27 (SST)  r = .12 (n-back)  r = .03 (OSPAN)  Fruit and vegetable consumption:  r = -.01 (Stroop)  r = -.03 (SST)  r = .20 (n-back)  r = .28 (OSPAN) | Potential |
| [Allom & Mullan (2012)](#_ENREF_5) | Prospective; n= 218 students (209 included in analysis), ages 16-45 (mean 20.06) | TOH | Fruit and vegetable consumption – Self-report | r = -.04 (TOH pre-planning time)  r = -.09 (TOH error) | Potential |

| Black et al. (2017) | Prospective, n=149 adults; mean age 25.89 | Stroop, Tower of London, operation  Span | Alcohol consumption – Self-report | Time 2 (2 weeks later) heavy episodic drinking^[[2]](#footnote-2)^:  r = .09 (Stroop ratio)  r = .10 (TOL items correct on first attempt)  r = .18 (TOL average plan time)  r = .13 (OSPAN) | Potential |
| --- | --- | --- | --- | --- | --- |
| [Bogg, Fukunaga, Finn, & Brown (2012)](#_ENREF_9) | Cross-sectional; n=27 students; ages 18-23 (mean 20.11) | Auditory Consonant Trigram test | Alcohol consumption – Self-report | r = -.01 | Potential |
| Bub et al. (2016) | Prospective, n=1,023 children; aged from 1 month to 15 years old | Children’s Stroop task, Child Behaviour Questionnaire (inhibitory control subscale) | Sleep – Self-report | Night wakings (8 years old):  r = .01 (Stroop)  r = -.07 (CBQ)  Night wakings (11 years old):  r = -.03 (Stroop)  r = -.06 (CBQ)  Sleepiness (8 years old):  r = -.05 (Stroop)  r = -.05 (CBQ)  Sleepiness (11 years old):  r = .02 (Stroop)  r = -.07 (CBQ)  Sleep problems (11 years old):  r = -.03 (Stroop)  r = -.09 (CBQ)  Sleep problems (15 years old):  r = -.03 (Stroop)  r = .003 (CBQ) | Potential |
| [Christiansen et al. (2012)](#_ENREF_13) | Cross-sectional, n=97 university staff and students; ages 18-59 (mean 28.95) | GNG | Alcohol consumption – Self-report | r = .09 (weekly alcohol consumption)  r = .04 (AUDIT) | Potential |
| [Colder & O'Connor (2002)](#_ENREF_15) | Retrospective; n=106 undergraduates; mean age 19.11 | GNG Task and Inhibitory Control Scale | Alcohol consumption – Self-report | r = .24 (GNG Commission errors)  r = .22 (GNG RT)  r = .28 (Inhibitory control scale) | Potential |
| Dvorak et al. (2016) | Prospective, n=74 college student drinkers; mean age 21.297 | Stop-signal task | Alcohol consumption – Self-report | Drinking days:  r = .09  Drinks consumed:  r = -.01 | Potential |
| [Ettenhofer et al. (2010)](#_ENREF_19) | Prospective observational study; n=91 HIV positive adults; mean age 42.25 | Executive functioning | Medication adherence - Objective | r = .33 | Potential |
| [Fadardi & Cox (2008)](#_ENREF_20) | Cross-sectional; n=87 students (mean age 22.19 (males), 24.13 (females)) | Stroop task | Alcohol consumption – Self-report | r =.05 | Potential |
| [Fernie et al. (2013)](#_ENREF_22) | Cross-lagged prospective; n= 287 adolescents; ages 12-13 (mean 13.33) | Stop-Signal task | Alcohol consumption – Self-report | r = .16 | Potential |
| [Fernie, Cole, Goudie, & Field (2010)](#_ENREF_21) | Correlational/retrospective; n=75 social drinkers from university students and staff (68 included in analyses); mean age 19.34 | GNG and Stop Signal Task | Alcohol consumption^[[3]](#footnote-3)^ - Self-report | r = -.17 (GNG inhibition errors & AUI)  r = -.03 (Stop inhibition errors & AUI) | Potential |

| [Friese et al. (2010)](#_ENREF_23) | Cross-sectional; n=60 males (49 included in analysis); ages 18-49 (mean age 25.20) | Complex span task | Alcohol consumption – Self-report | r =-.13 | Potential |
| --- | --- | --- | --- | --- | --- |
| [Giancola & Mezzich (2003)](#_ENREF_24) | Cross-sectional; n=340 female adolescents; ages 14-18 (mean age 16.1 (substance use disorder), 15.7 (controls)) | Porteus maze test, vigilance task, motor restraint task, Stroop task, block design test, picture arrangement test and object assembly test | Drug use – Self-report | r =.15 | Potential |
| [Goudriaan, Grekin, & Sher (2011)](#_ENREF_25) | Prospective; n=200 (176 retained at follow-up) college students; mean age 20.4 | GoStop task (Stop signal response inhibition task) | Alcohol consumption – Self-report | r =-.01 (Quantity/frequency)  r =-.10 (Heavy drinking) | Potential |
| [Hall (2012)](#_ENREF_26) | Prospective; n=208 adults; ages 18-89 (mean 45.21) | Stroop task and Go/No-go Task | Fatty food consumption – Self-report | r =.26 | Potential |
| [Hall et al. (2008b)](#_ENREF_31) | Prospective; Study 1: n=64 undergraduates; Study 2: n=124 undergraduates; mean age 19 | GNG^[[4]](#footnote-4)^ | Exercise and healthy dietary choice – Self-report | Study 1:  r = .19 (PAR T2 & RT overall)  Study 2:  r = .18 (NCI T2 & RT overall) | Potential |
| [Hall et al. (2006)](#_ENREF_28) | Cross-sectional; n=217 healthy adults; ages 20-100 (mean 54.90) | Stroop Task | Smoking, physical activity, and sleep difficulties (alcohol measure dismissed) – Self-report | r =-.24 (# of pack years smoked)  r =-.15 (# days exercise/week)  r =.04 (sleep difficulties) | Potential |

| [Harakeh et al. (2012)](#_ENREF_32) | Prospective cohort; n=2, 230 adolescents (1,816 included in time 3 assessment); mean age 16.3 | Memory-search task and a shifting-set task. | Smoking^[[5]](#footnote-5)^ - Self-report | r = .02 (working memory)  r = .01 (Inhibition)  r = .01 (Attentional flexibility) | Potential |
| --- | --- | --- | --- | --- | --- |
| [Henges & Marczinski (2012)](#_ENREF_33) | Cross-sectional; n=109 undergraduate students, ages 18-21 (mean 19.6) | Cued GNG | Alcohol consumption – Self-report | r = .22 (Total drinks & inhibition failures)  r = .23 (heavy drinking days & inhibition failures)  r = .19 (drunk days & inhibition failures)  r = .24 (highest number of drinks consumed in a day & inhibition failures) | Potential |
| [Hofmann et al. (2009)](#_ENREF_34) | Experimental; n=122 female students (118 included in analyses); mean age 23 | Operation Span Task, Stop Signal Paradigm | Candy consumption - Objective | r = .02 (Executive attention)  r = .06 (Inhibitory control) | Potential |
| [Hofmann et al. (2008)](#_ENREF_35) | Experimental; Study 2: n=119 (117 included in analyses) female undergraduates, ages 18-44 (mean 22.38) | Computation Span | Candy consumption - Objective | r = -.12 | Potential |
| [Houben & Wiers (2009)](#_ENREF_38) | Correlational; n=71; mean age 20.49 | Stroop Task | Alcohol consumption – Self-report | r = -.15 | Potential |
| [Ickmans et al. (2013)](#_ENREF_40) | Prospective case-controlled comparison between chronic fatigue syndrome patients and controls; n= 31 female patients and 13 healthy inactive females; 18-45 (mean age 35.6 & 29 respectively) | Stroop and operation span task | Physical activity - Objective | r =.40 (Ospan math error)  r = -.22 (OSPAN score)  r =.05 (Stroop RT Incongruent) | Low/no risk |
| [Jonker, Ostafin, Glashouwer, van Hemel-Ruiter, & de Jong (2014)](#_ENREF_41) | Cross-sectional; n=78 undergraduates (76 included in analysis), ages 18-32 | Attentional network task | Alcohol consumption – Self-report | r = -.06 | Potential |
| Khurana et al. (2015) | Prospective, n=382 adolescents, aged 11-13 years (mean 12.4) | Corsi block tapping, digit span backwards, letter two-back, and a spatial working memory task | Drug use, alcohol consumption and smoking^[[6]](#footnote-6)^ - Self-report | Drug use:  r = 0.05 (Corsi block tapping)  r = 0.04 (Digit span backwards)  r = 0.01 (Letter two-back)  r = 0.01 (Spatial working memory task)  Alcohol consumption:  r = 0.08 (Corsi block tapping)  r = 0.03 (Digit span backwards)  r = 0.04 (Letter two-back)  r = -0.01 (Spatial working memory task)  Smoking:  r = -0.01 (Corsi block tapping)  r = -0.01 (Digit span backwards)  r = -0.06 (Letter two-back)  r = -0.06 (Spatial working memory task) | Potential |
| Kim-Spoon et al. (2016) | Cross-sectional, n=157 early adolescents, aged 13-14 (mean 14.13) | Multi-Source Interference Task (MSIT) | Substance use (Drug use, alcohol consumption and smoking) – Self-report | Substance use severity:  r = -0.02 (MSIT behavioural factor score)  r = 0.08 (MSIT neural factor score)  Substance use onset:  r = -0.10 (MSIT behavioural factor score)  r = 0.06 (MSIT neural factor score) | Potential |

| [Kor & Mullan (2011)](#_ENREF_42) | Prospective study; n=273 psychology undergraduates (257 included in analyses); ages 16-56 (mean 19.9) | Visual GNG | Sleep hygiene behaviours – Self-report | r = .43 | Potential |
| --- | --- | --- | --- | --- | --- |
| [Larsen, Kong, Becker, Cousijn, Boendermaker, Cavallo et al. (2014)](#_ENREF_43) | Cross-sectional; 79 Dutch adolescents (41 smokers, 38 non-smokers) and 46 American adolescents (26 smokers, 20 non-smokers) (56 Dutch adolescents (27 smokers and 29 non-smokers) and 37 American adolescents (19 smokers and 18 non-smokers), ages 13-18 (mean age 16.08) | Stroop task and self-ordering pointing task | Smoking – Self-report | r = -.35 (Working memory)  r = -.01 (Inhibition skills) | Potential |
| [McAuley et al. (2011)](#_ENREF_48) | Prospective; n=177 older adults; ages 58-81(mean 66.44) | Dual task, Stroop colour-word task, Flanker task, WCST and task-switching | Physical activity adherence - Objective | r =.10 (Dual task % error)  r = -.01 (Stroop task cost)  r =.04 (Flanker proportional cost)  r = -.01 (WCST % per errors)  r =.02 (Task-switching cost) | Potential |
| Meisel et al. (2015) | Prospective, n= 363 adolescents, mean age 15.08 | Stop-signal task, and Tower of London | Alcohol consumption – Self-report | r = -0.06 (Wave 2^[[7]](#footnote-7)^ SSRT) (Response inhibition)  r = 0.05 (Wave 2 preplanning) (Planning) | Potential |
| [Menon, Jahn, Mauer, & O'Bryant (2013)](#_ENREF_49) | Cross-sectional; n=456 (cognitively impaired and cognitively intact), ages 40+ (mean age 62.40) | The Executive Interview | Alcohol consumption, smoking and cancer screening attendance – Self-report | r = -.12 (AUDIT)  r =.07 (Smoking)  r =.03 (Cancer screening) | Potential |
| [Mullan et al. (2011)](#_ENREF_52) | Prospective study; n=153 students; mean age 20.1 | TOH, Stroop Task, and the WCST | Binge-drinking – Self-report | r = .08 (TOH)  r = .09 (IGT)  r = .04 (WCST)  r = -.00 (Stroop) | Potential |
| [Murphy & Garavan (2011)](#_ENREF_53) | Retrospective; n=89 students consuming alcohol a minimum of once a week (84 included in analyses); ages 18-30 (mean 20.8) | GNG | Alcohol consumption – Self-report | r = .45 (commission errors) | Potential |
| [Patrick et al. (2008)](#_ENREF_56) | Correlational; n=80 students (72 females included in analysis), ages 19-24 (mean 21.09) | n-back Task and GNG | Alcohol consumption and drug use – Self-report | r = -.21 (n-back & alcohol)  r = .00 (GNG & alcohol)  r = -.15 (n-back & drugs)  r = -.10 (GNG & drugs) | Potential |

| Paz et al. (2016) | Prospective, n=49 undergraduates, aged 19-25 (mean 21.92) | Stop Signal task (SST), Go/No-Go task (GNG) and Simon task | Alcohol consumption^[[8]](#footnote-8)^ - Self-report | Total drinking days:  r = -0.21 (SSRT – N=49)  r = 0.05 (No-go errors – N=49)  r = 0.22 (Incongruent errors – N=49)  r = 0.05 (Simon effect - N=49)  Total binge days:  r = -0.16 (SSRT – N=49)  r = 0.09 (No-go errors – N=49)  r = 0.18 (Incongruent errors – N=49)  r = 0.07 (Simon effect - N=49)  Total intoxication days:  r = -0.09 (SSRT – N=49)  r = 0.41 (No-go errors – N=49)  r = 0.45 (Incongruent errors – N=49)  r = 0.08 (Simon effect - N=49)  AUQ binge score:  r = -0.08 (SSRT – N=49)  r = 0.23 (No-go errors – N=49)  r = 0.34 (Incongruent errors – N=49)  r = 0.06 (Simon effect - N=49)  AUDIT:  r = -0.50 (SSRT – N=49)  r = 0.19 (No-go errors – N=49)  r = 0.44 (Incongruent errors – N=49)  r = -0.02 (Simon effect - N=49) | Potential |
| --- | --- | --- | --- | --- | --- |

| Pentz et al. (2015) | Cross-sectional, n=410 7^th^ grade students, mean age 12.4 | Behavioral Inventory of Executive Function (BRIEF) | Alcohol consumption and smoking (cigarette and e-cigarette) – Self-report | Alcohol use (n=346):  r = 0.22  Cigarette use (n=347):  r = 0.17  e-cigarette use (n=346):  r = 0.16 | Potential |
| --- | --- | --- | --- | --- | --- |
| Pfeffer & Strobach (2017) | Prospective, n=118 graduate students, mean age 23.18 | Go/no-go task, stop-signal task, task-cueing paradigm, alternating-runs paradigm, N-back task, and visual memory task | Physical activity – Self-report | r = -0.08 (Go/no-go task)  r = -0.13 (SST)  r = 0.14 (Visual memory)  r = 0.19 (N-back)  r = -0.06 (Task-cueing)  r = 0.11 (Alternating-runs) | Potential |
| [Pharo et al (2011)](#_ENREF_57) | Cross-sectional; n=136 adolescents; ages 13-17 (mean 15.86) and n=57 young adults; ages 18-22 (mean 19.8) | COWAT, Mental Control, Backward Digit Span, Mental Arithmetic, WCST and the Stroop Test^[[9]](#footnote-9)^ | Alcohol, smoking and drug use – Self-report | r = .33 | Potential |
| [Pieters et al. (2014)](#_ENREF_58) | Longitudinal; n=427 adolescents; ages 12-16 (mean age13.96) | Self-ordered pointing task | Alcohol consumption – Self-report | r =.07 | Potential |
| [Pieters, Burk, Van der Vorst, Wiers, & Engels (2012)](#_ENREF_59) | Longitudinal, n=238 adolescents; mean age 13.82 | Self-ordered pointing task | Alcohol consumption – Self-report | r = .06 | Potential |

| Powell et al. (2017) | Prospective, n=64 adults, mean age 38.58 | Behavior Rating Inventory of  Executive Function—Adult version (BRIEF) and the Attention Switching Task, Stop Signal task and real time go/no-go task | Snack consumption^[[10]](#footnote-10)^ - Self-report | r = 0.12 (BRIEF)  r = 0.08 (SST)  r = -0.01 (GNG) | Potential |
| --- | --- | --- | --- | --- | --- |
| [Romer et al. (2011)](#_ENREF_61) | Longitudinal; n=387 (366 at time 3 assessment) children, ages 10-12 | Digit span backwards, Visual spatial working memory, Corsi block tapping, Letter two-back | Smoking and alcohol consumption – Self-report | r = .19^[[11]](#footnote-11)^ | Potential |
| [Romer, Betancourt, Giannetta, Brodsky, Farah, & Hurt (2009)](#_ENREF_60) | Multi-cohort longitudinal study; n=387 children; ages 10-12 | Corsi Block-Tapping, Letter Two-back, DS, Spatial Working Memory, Counting Stroop, Flanker Task | Smoking, alcohol consumption and drug use – Self-report | Alcohol:  r = -.00 (Two back)  r = -.00 (Digit span)  r = -.05 (Corsi)  r = -.06 (Spatial WM)  r = -.03 (Flanker)  r = -.10 (Stroop)  Smoking:  r = .04 (Two back)  r = -.07 (Digit span)  r = .00 (Corsi)  r = -.02 (Spatial WM)  r = -.11 (Flanker)  r = -.02 (Stroop) | Potential |
| [Salemink & Wiers (2014)](#_ENREF_65) | Prospective; n=92 (86 included in prospective drinking analyses); mean age 22.7 | Operation span task | Alcohol consumption – Self-report | r = -.11 (Total number of drinks next week)  r = -.02 (Number of binges next week) | Potential |
| [Sharbanee et al. (2013a)](#_ENREF_66) | Cross-sectional; n= 55 (27 light drinkers, 28 dysregulated drinkers) (48 (24 in each group) included in analysis); mean age 18.76 (light drinkers), 17.78 (dysregulated drinkers | Operation span task | Alcohol consumption – Self-report | r =.21 | Potential |
| [Sharbanee et al. (2013b)](#_ENREF_67) | Cross-sectional; n= 80 (40 problem, 40 nonproblem drinkers – 77 included in analysis); mean age 19.3 (problem drinkers), 19.7 (nonproblem drinkers) | Operation span task | Alcohol consumption – Self-report | r = -.14 | Potential |

| Stautz et al. (2016) | Prospective, n=6069 children, aged 8-13 | The Sky Search task and The Opposite Worlds task from the Test of Everyday Attention for Children (TEA-Ch), Counting Span Task and Stop Signal Task | Alcohol consumption, smoking, fruit and vegetable consumption – Self-report  Physical activity - Objective | Alcohol consumption (n= 3106):  r = 0.03 (Sky search task)  r = 0.04 (Opposite worlds task)  r = 0.04 (Counting span task)  r = -0.03 (SST)  Smoking (n=3106):  r = 0.00 (Sky search task)  r = -0.01 (Opposite worlds task)  r = -0.09 (Counting span task)  r = -0.01 (SST)  Fruit and vegetable consumption (n= 3099):  r = 0.02 (Sky search task)  r = 0.02 (Opposite worlds task)  r = 0.11 (Counting span task)  r = 0.00 (SST)  Physical activity (n= 2254):  r = -0.07 (Sky search task)  r = 0.00 (Opposite worlds task)  r = 0.00 (Counting span task)  r = 0.01 (SST) | Potential |
| --- | --- | --- | --- | --- | --- |
| [Stilley et al. (2010)](#_ENREF_69) | Study 1: longitudinal randomised control trial; n=157 patients being treated for hyperlipidemia, ages 24-60 (mean age 46.2)  Study 3: longitudinal; n=34 women with breast cancer; mean age 59.76 | Study 1: Trails B, digit span back  Study 3: Trails B,  CANTAB SWM, COWA, Delis Kaplan CWIT, CANTAB SOC | Medication adherence - Objective | Study 1:  r = -.17  Study 3:  r =.31 (# of prescribed doses)  r =.38 (% days with correct dose)  r =.23 (% days with optimal inter-dose interval) | Potential |
| [Stoltenberg, Batien, & Birgenheir (2008)](#_ENREF_72) | Cross-sectional; n=200; ages 18-47 (mean age 22.67) | Stop task | Tobacco use – Self-report | r =.00 (SSRT)  r =.05 (Accuracy for go trials) | Potential |
| [Todd & Mullan (2013)](#_ENREF_74) | Prospective; n= 190 students (137 included in analyses), mean age 19.7 | TOL, WCST, GNG and the Stroop task. | Sleep hygiene behaviours – Self-report | Behaviour 1 (making bedroom restful):  r = -.05 (GNG PI)  r = .13 (GNG Latency)  r = -.10 (IGT)  r =.03 (Stroop latency)  r = -.10 (Stroop score)  r = .12 (TOL FMT)  r = -.10 (TOL score)  r = -.04 (WCST)  Behaviour 2 (avoiding going to bed hungry or thirsty):  r = -.04 (GNG PI)  r = .03 (GNG Latency)  r = -.15 (IGT)  r = .09(Stroop latency)  r = .12 (Stroop score)  r = .08 (TOL FMT)  r = -.02 (TOL score)  r = -.26 (WCST)  Behaviour 3 (avoiding anxiety before bed):  r = -.03 (GNG PI)  r = .21 (GNG Latency)  r = -.12 (IGT)  r = -.11(Stroop latency)  r = .03 (Stroop score)  r = .04 (TOL FMT)  r = -.12 (TOL score)  r = -.03 (WCST) | Potential |
| Van Hemel-Ruiter et al. (2015) | Cross-sectional, n=86 adolescents, aged 12-18 (mean 14.86) | The Attention Network Task | Alcohol consumption – Self-report | r = 0.00 | Potential |

| [van Hemel-Ruiter, de Jong, & Wiers (2011)](#_ENREF_75) | Cross-sectional; n=55 adolescent drinkers (43 included in final analyses), ages 13-17 (mean age 15.09) | Random number generation task | Alcohol consumption – Self-report | r =.01 | Potential |
| --- | --- | --- | --- | --- | --- |
| Wardell et al. (2016) | Cross-sectional, n=300 young heavy episodic drinkers, aged 18- 25 (mean 19.75) | Digit span task | Alcohol consumption – Self-report | r = -0.10 (mean span)  r = -0.07 (two-error total)  r = -0.04 (median reaction time) | Potential |
| [Wong & Mullan (2009)](#_ENREF_79) | Prospective; n=96 psychology undergraduates, ages 17-30 (mean 19.46) | GNG and the TOH | Breakfast consumption – Self-report | r = .04 (GNG RT)  r = .04 (GNG PI)  r = .12 (TOH PT)  r = -.06 (TOH errors) | Potential |
| Wyckoff et al. (2017) | Prospective, n= 190 overweight and obese adults, aged 18-70 | D-KEFS Color-Word  Interference task and D-KEFS tower task | Fruit and vegetable consumption and saturated fat consumption^[[12]](#footnote-12)^ - Self-report | Saturated fat:  r = -0.00 (Planning – 1st move time)  r = -0.04 (Planning – rule violations)  r = 0.09 (Planning – Achievement score)  r = -0.14 (Inhibitory control)  Fruit and vegetable consumption:  r = 0.11 (Planning – 1st move time)  r = 0.19 (Planning – rule violations)  r = -0.06 (Planning – Achievement score)  r = -0.12 (Inhibitory control) | Potential |

*Note*: DEX = Dysexecutive Questionnaire, GNG = Go/No-go task, TOL = Tower of London, TOH = Tower of Hanoi, WCST = Wisconsin Card Sorting Task, SSRT = Stop Signal Reaction Time, RT = Reaction time, COWAT = Controlled Oral Word Association Test, AUI = Alcohol Use Index, AUDIT = Alcohol Use Disorders Identification Test, PAR T2 = Physical Activity Recall Time 2, NCI T2 = NCI Fruit & Vegetable Screener Time 2.

Supplementary Material 2: *Quality assessment criteria*

The risks of bias of included studies were assessed using the criteria below. For each criterion, a study was assessed as having a) no or low risk of bias, or b) potential risk of bias.

**For all studies**

Sampling:

1. Sample sizes are adequate (>=30).
2. If some participants were excluded from the analyses, the exclusion is justified.
3. When group comparisons are made, participants are matched on other meaningful demographics.

Measures (Executive function):

1. Validated measures are used, or the authors have provided sufficient supportive information of the psychometric properties of measures they devised.
2. Measures used were clearly defined and were appropriate.
3. Measures were objective rather than self-report.

Measures (Health behaviour):

1. Validated measures are used, or the authors have provided sufficient supportive information of the psychometric properties of measures they devised.
2. Measures used were clearly defined and were appropriate.
3. Measures were objective rather than self-report.

**For studies using longitudinal/prospective designs**

1. Dropout rates were not high (<20%), and missing data were treated appropriately

*Note.* + No or low risk of bias; − Potential risk of bias

Supplementary Material 3**:** *Studies Included in Meta-Analysis.*

Albertella, L., Copeland, J., Pearson, D., Watson, P., Wiers, R. W. & Le Pelley, M. E. (2017). Selective attention moderates the relationship between attentional capture by signals of nondrug reward and illicit drug use. Drug & Alcohol Dependence, 175, 99-105.

Allan, J. L., Johnston, M., & Campbell, N. (2010). Unintentional eating. What determines goal-incongruent chocolate consumption? *Appetite, 54*(2), 422-425.

Allan, J. L., Johnston, M., & Campbell, N. (2011). Missed by an inch or a mile? Predicting the size of intention-behaviour gap from measures of executive control. *Psychology & Health, 26*(6), 635-650.

Allan, J. L., Sniehotta, F. F., & Johnston, M. (2013). The Best Laid Plans: Planning Skill Determines the Effectiveness of Action Plans and Implementation Intentions. *Annals of Behavioral Medicine, 46*(1), 114-120.

Allom, V. & Mullan, B. (2014). Individual differences in executive function predict distinct eating behaviours. Appetite, 80, 123-130.

Allom, V., & Mullan, B. (2012). Self-regulation versus habit: The influence of self-schema on fruit and vegetable consumption. *Psychology & Health, 27*, 7-24.

Allom, V., Mullan, B., & Sebastian, J. (2013). Closing the intention-behaviour gap for sunscreen use and sun protection behaviours. *Psychology & Health, 28*(5), 477-494.

Black, N., Mullan, B. & Sharpe, L. (2017). Predicting heavy episodic drinking using an extended temporal self-regulation theory. Addictive Behaviors, 73, 111-118.

Bogg, T., Fukunaga, R., Finn, P. R., & Brown, J. W. (2012). Cognitive control links alcohol use, trait disinhibition, and reduced cognitive capacity: Evidence for medial prefrontal cortex dysregulation during reward-seeking behavior. *Drug and Alcohol Dependence, 122*(1-2), 112-118.

Bub, K. L., Robinson, L. E. & Curtis, D. S. (2016). Longitudinal Associations Between Self-Regulation and Health Across Childhood and Adolescence. Health Psychology, 35, 1235-1245.

Christiansen, P., Cole, J. C., Goudie, A. J., & Field, M. (2012). Components of behavioural impulsivity and automatic cue approach predict unique variance in hazardous drinking. *Psychopharmacology, 219*(2), 501-510.

Colder, C. R., & O'Connor, R. (2002). Attention bias and disinhibited behavior as predictors of alcohol use and enhancement reasons for drinking. *Psychology of Addictive Behaviors, 16*(4), 325-332.

Dvorak, R. D., Pearson, M. R., Sargent, E. M., Stevenson, B. L. & Mfon, A. M. (2016). Daily associations between emotional functioning and alcohol involvement: Moderating effects of response inhibition and gender. Drug and Alcohol Dependence, 163, S46-S53.

Ettenhofer, M. L., Foley, J., Castellon, S. A., & Hinkin, C. H. (2010). Reciprocal prediction of medication adherence and neurocognition in HIV/AIDS. *Neurology, 74*(15), 1217-1222.

Fadardi, J. S., & Cox, W. (2008). Alcohol-attentional bias and motivational structure as independent predictors of social drinkers' alcohol consumption. *Drug and Alcohol Dependence, 97*(3), 247-256.

Fernie, G., Cole, J. C., Goudie, A. J., & Field, M. (2010). Risk-taking but not response inhibition or delay discounting predict alcohol consumption in social drinkers. *Drug and Alcohol Dependence, 112*(1-2), 54-61.

Fernie, G., Peeters, M., Gullo, M. J., Christiansen, P., Cole, J. C., Sumnall, H., et al. (2013). Multiple behavioural impulsivity tasks predict prospective alcohol involvement in adolescents. *Addiction, 108*(11), 1916-1923.

Friese, M., Bargas-Avila, J., Hofmann, W., & Wiers, R. W. (2010). Here's looking at you, Bud: Alcohol-related memory structures predict eye movements for social drinkers with low executive control. *Social Psychological and Personality Science, 1*(2), 143-151.

Giancola, P. R., & Mezzich, A. C. (2003). Executive functioning, temperament, and drug use involvement in adolescent females with a substance use disorder. *Journal of Child Psychology and Psychiatry and Allied Disciplines, 44*(6), 857-866.

Goudriaan, A. E., Grekin, E. R., & Sher, K. J. (2011). Decision making and response inhibition as predictors of heavy alcohol use: A prospective study. *Alcoholism: Clinical and Experimental Research, 35*(6), 1050-1057.

Hall, P. A. (2012). Executive Control Resources and Frequency of Fatty Food Consumption: Findings From an Age-Stratified Community Sample. *Health Psychology, 31*(2), 235-241.

Hall, P. A., Elias, L. J., & Crossley, M. (2006). Neurocognitive influences on health behavior in a community sample. *Health Psychology, 25*(6), 778-782.

Hall, P. A., Fong, G. T., Epp, L. J., & Elias, L. J. (2008b). Executive function moderates the intention-behavior link for physical activity and dietary behavior. *Psychology & Health, 23*(3), 309-326.

Harakeh, Z., de Sonneville, L., van den Eijnden, R. J. J. M., Huizink, A. C., Reijneveld, S. A., Ormel, J., et al. (2012). The Association Between Neurocognitive Functioning and Smoking in Adolescence: The TRAILS Study. *Neuropsychology, 26*(5), 541-550.

Henges, A. L., & Marczinski, C. A. (2012). Impulsivity and alcohol consumption in young social drinkers. *Addictive Behaviors, 37*(2), 217-220.

Hofmann, W., Friese, M., & Roefs, A. (2009). Three ways to resist temptation: The independent contributions of executive attention, inhibitory control, and affect regulation to the impulse control of eating behavior. *Journal of Experimental Social Psychology, 45*(2), 431-435.

Hofmann, W., Gschwendner, T., Friese, M., Wiers, R. W., & Schmitt, M. (2008). Working memory capacity and self-regulatory behavior: Toward an individual differences perspective on behavior determination by automatic versus controlled processes. *Journal of Personality and Social Psychology, 95*(4), 962-977.

Ickmans, K., Clarys, P., Nijs, J., Meeus, M., Aerenhouts, D., Zinzen, E., et al. (2013). Association between cognitive performance, physical fitness, and physical activity level in women with chronic fatigue syndrome. *Journal of Rehabilitation Research & Development, 50*(6), 795-810.

Jonker, N. C., Ostafin, B. D., Glashouwer, K. A., van Hemel-Ruiter, M. E., & de Jong, P. J. (2014). Reward and punishment sensitivity and alcohol use: The moderating role of executive control. *Addictive Behaviors, 39*(5), 945-948.

Khurana, A., Romer, D., Betancourt, L. M., Brodsky, N. L., Giannetta, J. M. & Hurt, H. (2015). Experimentation versus progression in adolescent drug use: A test of an emerging neurobehavioral imbalance model. Development & Psychopathology, 27, 901-13.

Kim-Spoon, J., Deater-Deckard, K., Holmes, C., Lee, J., Chiu, P. & King-Casas, B. (2016). Behavioral and neural inhibitory control moderates the effects of reward sensitivity on adolescent substance use. Neuropsychologia, 91, 318-326.

Kor, K., & Mullan, B. A. (2011). Sleep hygiene behaviours: An application of the theory of planned behaviour and the investigation of perceived autonomy support, past behaviour and response inhibition. *Psychology & Health, 26*(9), 1208-1224.

Larsen, H., Kong, G., Becker, D., Cousijn, J., Boendermaker, W., Cavallo, D., et al. (2014). Implicit motivational processes underlying smoking in American and Dutch adolescents. *Frontiers in Psychiatry, 5*(MAY).

McAuley, E., Mullen, S. P., Szabo, A. N., White, S. M., Wojcicki, T. R., Mailey, E. L., et al. (2011). Self-regulatory processes and exercise adherence in older adults: Executive function and self-efficacy effects. *American Journal of Preventive Medicine, 41*(3), 284-290.

Meisel, S. N., Colder, C. R. & Hawk, L. W. (2015). The moderating role of cognitive capacities in the association between social norms and drinking behaviors. Alcoholism: Clinical & Experimental Research, 39, 1049-56.

Menon, C. V., Jahn, D. R., Mauer, C. B., & O'Bryant, S. E. (2013). Executive functioning as a mediator of the relationship between premorbid verbal intelligence and health risk behaviors in a rural-dwelling cohort: a Project FRONTIER study. *Archives of Clinical Neuropsychology, 28*(2), 169-179.

Mullan, B., Wong, C., Allom, V., & Pack, S. L. (2011). The role of executive function in bridging the intention-behaviour gap for binge-drinking in university students. *Addictive Behaviors, 36*(10), 1023-1026.

Murphy, P., & Garavan, H. (2011). Cognitive predictors of problem drinking and AUDIT scores among college students. *Drug and Alcohol Dependence, 115*(1-2), 94-100.

Patrick, M. E., Blair, C., & Maggs, J. L. (2008). Executive function, approach sensitivity, and emotional decision making as influences on risk behaviors in young adults. *Journal of Clinical and Experimental Neuropsychology, 30*(4), 449-462.

Paz, A. L., Keim, C. A. & Rosselli, M. (2016). Inhibitory performance predicting drinking behaviours among young adults. Alcohol and Alcoholism, 51, 677-683.

Pentz, M. A., Shin, H., Riggs, N., Unger, J. B., Collison, K. L. & Chou, C. P. (2015). Parent, peer, and executive function relationships to early adolescent e-cigarette use: a substance use pathway? Addictive Behaviors, 42, 73-8.

Pfeffer, I. & Strobach, T. (2017). Executive functions, trait self-control, and the intention-behavior gap in physical activity behavior. Journal of Sport & Exercise Psychology, 39, 277-292.

Pharo, H., Sim, C., Graham, M., Gross, J., & Hayne, H. (2011). Risky business: Executive function, personality, and reckless behavior during adolescence and emerging adulthood. *Behavioral Neuroscience, 125*(6), 970-978.

Pieters, S., Burk, W. J., Van der Vorst, H., Engels, R. C., & Wiers, R. W. (2014). Impulsive and reflective processes related to alcohol use in young adolescents. *Frontiers in Psychiatry, 5*.

Pieters, S., Burk, W. J., Van der Vorst, H., Wiers, R. W., & Engels, R. C. M. E. (2012). The Moderating Role of Working Memory Capacity and Alcohol-Specific Rule-Setting on the Relation Between Approach Tendencies and Alcohol Use in Young Adolescents. *Alcoholism-Clinical and Experimental Research, 36*(5), 915-922.

Powell, D. J., Mcminn, D. & Allan, J. L. (2017). Does real time variability in inhibitory control drive snacking behavior? An intensive longitudinal study. Health Psychology, 36, 356-364.

Romer, D., Betancourt, L., Giannetta, J. M., Brodsky, N. L., Farah, M., & Hurt, H. (2009). Executive cognitive functions and impulsivity as correlates of risk taking and problem behavior in preadolescents. *Neuropsychologia, 47*(13), 2916-2926.

Romer, D., Betancourt, L. M., Brodsky, N. L., Giannetta, J. M., Yang, W., & Hurt, H. (2011). Does adolescent risk taking imply weak executive function? A prospective study of relations between working memory performance, impulsivity, and risk taking in early adolescence. *Developmental Science, 14*(5), 1119-1133.

Salemink, E., & Wiers, R. W. (2014). Alcohol-related memory associations in positive and negative affect situations: Drinking motives, working memory capacity, and prospective drinking. *Psychology of Addictive Behaviors, 28*(1), 105-113.

Sharbanee, J. M., Stritzke, W. G., Wiers, R. W., & MacLeod, C. (2013a). Alcohol-related biases in selective attention and action tendency make distinct contributions to dysregulated drinking behaviour. *Addiction, 108*(10), 1758-1766.

Sharbanee, J. M., Stritzke, W. G., Wiers, R. W., Young, P., Rinck, M., & MacLeod, C. (2013b). The interaction of approach-alcohol action tendencies, working memory capacity, and current task goals predicts the inability to regulate drinking behavior. *Psychology of Addictive Behaviors, 27*(3), 649-661.

Stautz, K., Pechey, R., Couturier, D. L., Deary, I. J. & Marteau, T. M. (2016). Do Executive Function and Impulsivity Predict Adolescent Health Behaviour after Accounting for Intelligence? Findings from the ALSPAC Cohort. PLoS ONE, 11, e0160512.

Stilley, C. S., Bender, C. M., Dunbar-Jacob, J., Sereika, S., & Ryan, C. M. (2010). The impact of cognitive function on medication management: three studies. *Health Psychology, 29*(1), 50-55.

Stoltenberg, S. F., Batien, B. D., & Birgenheir, D. G. (2008). Does gender moderate associations among impulsivity and health-risk behaviors? *Addictive Behaviors, 33*(2), 252-265.

Todd, J., & Mullan, B. (2013). The role of self-regulation in predicting sleep hygiene in university students. *Psychology Health & Medicine, 18*(3), 275-288.

Van Hemel-Ruiter, M. E., De Jong, P. J., Ostafin, B. D. & Wiers, R. W. (2015). Reward sensitivity, attentional bias, and executive control in early adolescent alcohol use. Addictive Behaviors, 40, 84-90.

van Hemel-Ruiter, M. E., de Jong, P. J., & Wiers, R. W. (2011). Appetitive and regulatory processes in young adolescent drinkers. *Addictive Behaviors, 36*(1-2), 18-26.

Wardell, J. D., Quilty, L. C. & Hendershot, C. S. (2016). Impulsivity, working memory, and impaired control over alcohol: A latent variable analysis. Psychology of Addictive Behaviors, 30, 544-554.

Wong, C. L., & Mullan, B. A. (2009). Predicting breakfast consumption: An application of the theory of planned behaviour and the investigation of past behaviour and executive function. *British Journal of Health Psychology, 14*(3), 489-504.

Wyckoff, E. P., Evans, B. C., Manasse, S. M., Butryn, M. L. & Forman, E. M. (2017). Executive functioning and dietary intake: Neurocognitive correlates of fruit, vegetable, and saturated fat intake in adults with obesity. Appetite, 111, 79-85.

Supplementary Material 4**:** *Search Strategy: PsycINFO (OVID) – Updated 2018 search*

1. ((executive control or executive function* or executive cognitive function or cognitive control or inhibition or working memory) not mental illness not brain injury).id

2. limit 1 to (peer reviewed journal and human and english language and yr="2014 -Current")

3. ((health behavio?r* or medication adherence or exercise or physical activity or sleep or eating or food consumption or alcohol or substance abuse or smoking) not mental illness not brain injury).id.

4. limit 2 to (peer reviewed journal and human and english language and yr="2014 -Current")

5. 1 and 3

6. limit 5 to (peer reviewed journal and human and english language)

7. limit 5 to (peer reviewed journal and human and english language and yr="2014 -Current")

| **Section/topic** | **#** | **Checklist item** | **Reported on page #** |
| --- | --- | --- | --- |
| **TITLE** | | |  |
| Title | 1 | Identify the report as a systematic review, meta-analysis, or both. | 1 |
| **ABSTRACT** | | |  |
| Structured summary | 2 | Provide a structured summary including, as applicable: background; objectives; data sources; study eligibility criteria, participants, and interventions; study appraisal and synthesis methods; results; limitations; conclusions and implications of key findings; systematic review registration number. | 2 |
| **INTRODUCTION** | | |  |
| Rationale | 3 | Describe the rationale for the review in the context of what is already known. | 3-7 |
| Objectives | 4 | Provide an explicit statement of questions being addressed with reference to participants, interventions, comparisons, outcomes, and study design (PICOS). | 3-7 |
| **METHODS** | | |  |
| Protocol and registration | 5 | Indicate if a review protocol exists, if and where it can be accessed (e.g., Web address), and, if available, provide registration information including registration number. | n/a |
| Eligibility criteria | 6 | Specify study characteristics (e.g., PICOS, length of follow-up) and report characteristics (e.g., years considered, language, publication status) used as criteria for eligibility, giving rationale. | 7-12 |
| Information sources | 7 | Describe all information sources (e.g., databases with dates of coverage, contact with study authors to identify additional studies) in the search and date last searched. | 7-12 |
| Search | 8 | Present full electronic search strategy for at least one database, including any limits used, such that it could be repeated. | 7-8 |
| Study selection | 9 | State the process for selecting studies (i.e., screening, eligibility, included in systematic review, and, if applicable, included in the meta-analysis). | 7-12 |
| Data collection process | 10 | Describe method of data extraction from reports (e.g., piloted forms, independently, in duplicate) and any processes for obtaining and confirming data from investigators. | 7-12 |
| Data items | 11 | List and define all variables for which data were sought (e.g., PICOS, funding sources) and any assumptions and simplifications made. | 7-12 |
| Risk of bias in individual studies | 12 | Describe methods used for assessing risk of bias of individual studies (including specification of whether this was done at the study or outcome level), and how this information is to be used in any data synthesis. | 7-12 |
| Summary measures | 13 | State the principal summary measures (e.g., risk ratio, difference in means). | 7-12 |
| Synthesis of results | 14 | Describe the methods of handling data and combining results of studies, if done, including measures of consistency (e.g., I^2^) for each meta-analysis. | 7-12 |

Page 1 of 2

| **Section/topic** | **#** | **Checklist item** | **Reported on page #** |
| --- | --- | --- | --- |
| Risk of bias across studies | 15 | Specify any assessment of risk of bias that may affect the cumulative evidence (e.g., publication bias, selective reporting within studies). | 7-12 |
| Additional analyses | 16 | Describe methods of additional analyses (e.g., sensitivity or subgroup analyses, meta-regression), if done, indicating which were pre-specified. | 7-12 |
| **RESULTS** | | |  |
| Study selection | 17 | Give numbers of studies screened, assessed for eligibility, and included in the review, with reasons for exclusions at each stage, ideally with a flow diagram. | Figure 1 |
| Study characteristics | 18 | For each study, present characteristics for which data were extracted (e.g., study size, PICOS, follow-up period) and provide the citations. | Supplementary Table 1 |
| Risk of bias within studies | 19 | Present data on risk of bias of each study and, if available, any outcome level assessment (see item 12). | Supplementary Table 2 |
| Results of individual studies | 20 | For all outcomes considered (benefits or harms), present, for each study: (a) simple summary data for each intervention group (b) effect estimates and confidence intervals, ideally with a forest plot. | 12-15 |
| Synthesis of results | 21 | Present results of each meta-analysis done, including confidence intervals and measures of consistency. | 12-15 |
| Risk of bias across studies | 22 | Present results of any assessment of risk of bias across studies (see Item 15). | 12-15 |
| Additional analysis | 23 | Give results of additional analyses, if done (e.g., sensitivity or subgroup analyses, meta-regression [see Item 16]). | 12-15 |
| **DISCUSSION** | | |  |
| Summary of evidence | 24 | Summarize the main findings including the strength of evidence for each main outcome; consider their relevance to key groups (e.g., healthcare providers, users, and policy makers). | 15-18 |
| Limitations | 25 | Discuss limitations at study and outcome level (e.g., risk of bias), and at review-level (e.g., incomplete retrieval of identified research, reporting bias). | 15-18 |
| Conclusions | 26 | Provide a general interpretation of the results in the context of other evidence, and implications for future research. | 15-18 |
| **FUNDING** | | |  |
| Funding | 27 | Describe sources of funding for the systematic review and other support (e.g., supply of data); role of funders for the systematic review. | n/a |

*From:*  Moher D, Liberati A, Tetzlaff J, Altman DG, The PRISMA Group (2009). Preferred Reporting Items for Systematic Reviews and Meta-Analyses: The PRISMA Statement. PLoS Med 6(6): e1000097. doi:10.1371/journal.pmed1000097

For more information, visit: **www.prisma-statement.org**.

Page 2 of 2

1. Rounded to two decimal places [↑](#footnote-ref-1)
2. Time 1 heavy episodic drinking correlations excluded [↑](#footnote-ref-2)
3. Alcohol Use Index used in analysis rather than separate alcohol measures. [↑](#footnote-ref-3)
4. Due to the reaction times for Go and No-go trials being moderately correlated the authors created an overall reaction variable, which was used in the meta-analysis. [↑](#footnote-ref-4)
5. Only daily smoking correlations included in meta-analysis. Smoking onset correlations dismissed. [↑](#footnote-ref-5)
6. Only Time 4 behavioural assessments included in analysis. [↑](#footnote-ref-6)
7. Only correlations between Wave 2 measures of executive function and Wave 4 measures of alcohol consumption included. [↑](#footnote-ref-7)
8. Total hangover days, age of first drink and age of first binge session excluded from analysis. [↑](#footnote-ref-8)
9. Composite neuropsychological functioning measure used in meta-analysis. [↑](#footnote-ref-9)
10. Correlations with baseline snack consumption excluded. [↑](#footnote-ref-10)
11. Working memory at time 2 correlated with risk behaviour at time 3 [↑](#footnote-ref-11)
12. Overall dietary intake (i.e., calories) not included. [↑](#footnote-ref-12)
